# Oral contraceptives and tissue factor expression. Comments on “Do combined oral contraceptives induce formation of tissue factor?”

**DOI:** 10.1016/j.rpth.2026.103445

**Published:** 2026-03-26

**Authors:** Bjarne Østerud

**Affiliations:** Department of Medical Biology, The Health Faculty, UiT The Arctic University of Norway

To the Editor,

Kristensen et al. [1] present a well-designed paired study evaluating whether combined oral contraceptives (COCs) alter extracellular vesicle (EV)–associated tissue factor (TF), procoagulant phospholipids, or EV concentration [1]. Their finding that second-generation COCs do not increase circulating EV-TF or procoagulant phospholipids adds important data to the understanding of COC-related hemostatic changes.

However, the discussion relies on earlier *in vitro* studies suggesting that estrogen or COCs may activate endothelial cells or monocytes and thereby increase TF expression. This interpretation does not reflect the substantial *in vivo* evidence demonstrating that physiological estrogen exposure suppresses monocyte TF activity and reduces inflammatory responsiveness.

Across several studies, we have shown that estrogen exposure, whether through oral contraceptives, pregnancy, or hormone replacement therapy, reduces monocyte TF activity and attenuates lipopolysaccharide-induced TF responses. In oral contraceptive users, monocyte TF activity is reduced rather than increased [2]. In pregnant women, monocytes show both reduced basal TF activity and reduced sensitivity to inflammatory stimulation [3]. Similarly, hormone replacement therapy decreases monocyte and platelet reactivity in whole blood [4]. These findings are supported by work from other groups, demonstrating estrogen’s anti-inflammatory effects on monocytes and vascular cells [[5], [6], [7]].

In our view, the discrepancy between earlier *in vitro* reports of estrogen-induced TF upregulation and the consistent *in vivo* suppression of monocyte TF activity is best explained by methodological factors. Monocytes placed into cell culture undergo rapid preactivation, resulting in markedly higher TF expression compared with freshly isolated *ex vivo* monocytes [8]. Under these conditions, even weak stimuli can further enhance TF expression, creating an artificial impression of estrogen-induced activation. In addition, test compounds used in cell culture, including steroid hormones, are frequently contaminated with trace amounts of endotoxin unless specifically detoxified. Such contamination is sufficient to induce or amplify TF expression in preactivated monocytes. These factors together provide a more plausible explanation for the *in vitro* findings than any true physiological proinflammatory effect of estrogen. *In vivo*, where monocytes remain in their native, nonactivated state and are not exposed to endotoxin-contaminated reagents, estrogen consistently exerts a suppressive effect on TF induction.

A brief methodological comment concerns the interpretation of the very low TF activities detected in isolated EV fractions. Although the authors have developed a sensitive assay, distinguishing true TF activity from phospholipid-dependent enhancement of thrombin generation at such low levels remains challenging. Several groups have reported difficulty detecting TF in EVs from healthy individuals even with highly sensitive methods [9]. While the authors state that their assay is specific, the possibility that the small activity detected reflects residual phospholipid-related signal cannot be fully excluded.

The findings by Kristensen et al. [1] are consistent with the broader *in vivo* literature, demonstrating that estrogen does not enhance monocyte TF activity and may, in fact, suppress TF responses to inflammatory stimuli. Their results reinforce that TF-bearing EVs are unlikely to contribute meaningfully to COC-associated thrombotic risk.
